# Olanzapine 5 mg for Nausea and Vomiting in Patients with Nasopharyngeal Carcinoma Receiving Cisplatin-Based Concurrent Chemoradiotherapy

**DOI:** 10.1155/2022/9984738

**Published:** 2022-03-21

**Authors:** Jun Wang, Tao Li, Li-E Lin, Qin Lin, San-Gang Wu

**Affiliations:** ^1^Department of Head and Neck Oncology, Department of Radiation Oncology, Cancer Center, State Key Laboratory of Biotherapy, West China Hospital, Sichuan University, Chengdu 610041, China; ^2^Department of Radiation Oncology, Xiamen Cancer Center, Xiamen Key Laboratory of Radiation Oncology, The First Affiliated Hospital of Xiamen University, School of Medicine, Xiamen University, Xiamen 361003, China

## Abstract

**Purpose:**

To explore the efficacy and safety of adding olanzapine (5 mg or 10 mg) to 5-hydroxytryptamine type 3 receptor antagonists (5-HT_3_ RA), neurokinin-1 receptor antagonists (NK1 RA), and dexamethasone for nausea and vomiting in patients with nasopharyngeal carcinoma (NPC) receiving cisplatin-based concurrent chemoradiotherapy.

**Methods:**

Patients receiving olanzapine 5 mg or 10 mg combined with 5-HT_3_ RA, NK1 RA, and dexamethasone during the cisplatin-based concurrent chemoradiotherapy were included. The primary endpoint was the complete response (CR) (no vomiting) rate, and the secondary endpoint was the incidence of no nausea.

**Results:**

A total of 150 chemotherapy cycles were administrated for 88 patients (75 in the olanzapine 5 mg group and 75 in the olanzapine 10 mg group). The proportions of CR in the olanzapine 5 mg group were comparable to those in the olanzapine 10 mg group in acute (93.3% vs. 94.7%, *P* = 0.731), delayed (76% vs. 78.7%, *P* = 0.697), and overall phase (73.3% vs. 77.3%, *P* = 0.570). Moreover, no nausea rates were also comparable between the two groups in acute (76% vs. 78.7%, *P* = 0.697), delayed (54.7% vs. 60%, *P* = 0.509), and overall period (50.7% vs. 57.3%, *P* = 0.111). Regarding the adverse effects, the incidence of somnolence in the 10 mg group (58.6%) was significantly higher than that in the 5 mg group (41.3%) (*P* = 0.034), while constipation (20.0% vs. 24.0%, *P* = 0.554) and hiccups (9.3% vs. 10.6%, *P* = 0.785) rates were comparable in two groups.

**Conclusions:**

Patients receiving olanzapine plus standard antiemetic therapy has excellent antiemetic effect in NPC patients receiving cisplatin-based concurrent chemoradiotherapy, and patients with olanzapine 5 mg have a similar antiemetic effect and lower adverse effects compared with those with olanzapine 10 mg.

## 1. Introduction

Nasopharyngeal carcinoma (NPC) is a subtype of head and neck malignant tumor characterized by unique geographical distribution in Southern China, Southeast Asia, and North Africa [1]. Approximately 70-80% of the NPC patients are diagnosed with stage III-IVA disease, and guidelines for current clinical practice recommend that platinum-based induction chemotherapy (IC) + concurrent chemoradiotherapy (CCRT) or platinum-based CCRT + adjuvant chemotherapy serves as the standard management in this disease, with a 5-year overall survival rate about 85% [2–4].

Cisplatin, as a highly emetogenic drug, is recommended for a total dose of 100 mg/m^2^ triweekly or 40 mg/m^2^ weekly in patients with NPC receiving CCRT [3]. However, several studies demonstrated that patients receiving a standard dose of cisplatin had higher adverse effects, especially gastrointestinal reactions and hematological toxicities, and that approximately 6%-16% of the patients uncompleted the scheduled courses or reduced the dose of chemotherapy, severely affecting the therapeutic effect [5–7]. Chemotherapy-induced nausea and vomiting (CINV), as a common adverse effect of cisplatin, with an estimated prevalence of 70–80%, seriously affect the quality of life, treatment compliance, and even therapeutic efficacy of the patients with cancer, including NPC [8–10]. A previous study has found that 35.7% of the NPC patients receiving CCRT suffered CINV [11]. The international guidelines established by the American Society of Clinical Oncology, Multinational Association of Supportive Care in Cancer, and the European Society of Medical Oncology have recommended a combination of olanzapine 10 mg with 5-hydroxytryptamine type 3 receptor antagonists (5-HT_3_ RA), neurokinin-1 receptor antagonists (NK_1_ RA), and dexamethasone against CINV in cancer patients receiving highly emetogenic chemotherapy, while olanzapine 5-10 mg was suggested in National Comprehensive Cancer Network guideline [12–14].

Olanzapine is an atypical antipsychotic drug that blocks serotonin receptors (5HT_2a_, 5HT_2c_, 5HT_3_, 5-HT_6_), dopamine receptors (D_1_, D_2_, D_4_), histamine H1 receptors, *α*1 adrenergic receptors, and muscarinic receptors [15–17]. Therefore, the US Food and Drug Administration (FDA) has approved it for the treatment of psychotic disturbance [15]. D_2_ dopamine receptor and 5HT_3_ serotonin receptor appear to correlate with nausea and vomiting; thus, olanzapine is also applied for the prevention of CINV in cancer patients receiving highly emetogenic chemotherapy [18]. Nevertheless, olanzapine still has inevitable adverse effects, including somnolence, constipation, dry mouth, and hiccups [19]. To our knowledge, there is no study exploring the efficacy and safety of additional olanzapine to standard triplet-combination in NPC patients receiving CCRT. Therefore, we aim to explore the effect of olanzapine 5 mg or 10 mg plus triple combination on the control of CCRT-induced nausea and vomiting.

## 2. Materials and Methods

### 2.1. Patient Selection

The data was prospectively collected from the Department of Radiation Oncology, the First Affiliated Hospital of Xiamen University between Apr 2018 and May 2019. Patients meeting the following criteria were eligible: (1) pathologically diagnosed with NPC; (2) receiving cisplatin-based CCRT; (3) not using the drugs that affect the result of the study, such as 5-HT_3_ RA, NK_1_ RA, and corticosteroids, within 48 h before treatment; (4) Eastern Cooperative Oncology Group performance status of 0–2; and (5) providing written informed consents. Patients not receiving CCRT or receiving noncisplatin CCRT regimens were excluded. We also excluded the patients with the following severe comorbidities: unstable angina, myocardial infarction, cerebral infarction, or allergic to olanzapine, which might be intolerable to olanzapine. TNM staging was evaluated using the 8th edition American Joint Committee on Cancer/Union for International Cancer Control staging system (T-tumor, N-node, M-metastasis). This study was approved by the ethics committee of the First Affiliated Hospital of Xiamen University (Approved number: XMYY-2021KY053).

### 2.2. Treatment

A total of 150 chemotherapy cycles were administrated for 88 NPC patients between Apr 2018 and May 2019. Of the patients, 70.5% (*n* = 62) received 2-3 cycles of IC, including TPF (docetaxel, cisplatin, and 5-fluorouracil), TP, or GP (gemcitabine, cisplatin) regimens. Then patients were scheduled to receive CCRT within 3-4 weeks from the initiation of the last cycle of IC. Cisplatin 100 mg/m^2^ was given intravenously on days 1 (the day radiotherapy began) and 22 (the day irradiating for about 16 times) concurrently with radiotherapy. Intensity-modulated radiotherapy technique was used for radiotherapy with a prescribed dose of gross target volume: 70 grays (Gy) in 32 fractions to the nasopharynx and 66 Gy in 32 fractions to the neck lymph nodes, 5 days per week, for 6-7 weeks.

All patients received a four-drug combination antiemetic regimen consisting of tropisetron (5-HT_3_ RA), aprepitant (NK_1_ RA), dexamethasone, and olanzapine. Tropisetron 5 mg was administered 30-60 minutes before chemotherapy (intravenously on day 1, orally on days 2-3). Aprepitant was orally administered 60-90 minutes before chemotherapy (125 mg on day 1, 80 mg on days 2-3). Dexamethasone 10 mg was intravenously administered 30-60 minutes before chemotherapy on days 1-3. Olanzapine was randomly administered orally at a dose of 5 mg or 10 mg once per night on days 1-5. The participants could be recruited repeatedly during any CCRT cycles.

### 2.3. Assessment Procedure

The primary endpoint of this study was complete response (CR) (no vomiting) rate, defined as no emesis and rescue medication in acute (0-24 hours after the initiation of cisplatin), delayed (24-120 hours after the initiation of cisplatin), and overall phase (0 to 120 hours after the initiation of cisplatin). The secondary endpoint was the incidence of no nausea, defined as a visual analog scale (VAS) of 0 in acute, delayed, and overall phases.

The data of daily episodes of vomiting, degrees of nausea, and use of antiemetic drugs were collected from medical records from the initiation of cisplatin (day 1) to day 5. Degrees of nausea were daily assessed using VAS (scale ranging from 0 to 10, with 0 for no nausea and 10 for the maximal level of nausea). The adverse effects were evaluated by investigators and patients, according to the National Cancer Institute's Common Terminology Criteria for Adverse Events (CTCAE) version 4.0 (available at https://ctep.cancer.gov/protocolDevelopment/electronic_applications/ctc.htm).

### 2.4. Statistical Analysis

Chi-square test was applied to compare possible differences of CR and no nausea rates in the acute, delayed, and overall period between olanzapine 5 mg and 10 mg groups. Excel was used to paint the histogram for the percentage of CR and no nausea from day 1 to 5. The percentages of antiemetic-related adverse effects were computed according to the CTCAE version 4.0. IBM SPSS (version 22.0, IBM Corporation, Armonk, NY, USA) was used for statistical analysis, and a *P* < 0.05 was defined as significant.

## 3. Results

### 3.1. Patient Clinicopathological Characteristics

A total of 88 NPC patients were included in this study. Of these patients, 62 were enrolled at the beginning of the first and the second cycle of cisplatin-based CCRT. In addition, 26 patients were enrolled in the second cycle of cisplatin-based CCRT because they had completed the first cycle of cisplatin-based CCRT prior to the start of the study. Therefore, a total of 150 chemotherapy cycles were administrated in this study. The receipt of olanzapine 5 mg or 10 mg was randomized in each chemotherapy cycle. 5 mg oral olanzapine was administrated in 75 chemotherapy cycles, and 10 mg oral olanzapine was also administrated in 75 chemotherapy cycles.  Table 1 shows the detailed information of the study population. The mean age of the patients was 46 years (range, 23-64 years). Male and female patients were accounting for 77.3% (*n* = 68) and 22.7% (*n* = 20), respectively. The percentages of patients with smoking history and alcohol consumption were 56.8% and 25%, respectively. In addition, 76 (86.4%), 9 (10.2%), and 3 (3.4%) of the patients had pathological types of undifferentiated nonkeratinizing NPC, differentiated nonkeratinizing NPC, and the mixture of the above two. The majority of patients had T2-3 (68.2%, *n* = 60), N1-2 (69.3%, *n* = 61), and M0 stage (94.3%, *n* = 83). A total of 14 (15.9%), 36 (40.9%), 33 (37.5%), and 5 (5.7%) patients had stage II, III, IVa, and IVb disease.

### 3.2. Effects

**Figure 1:**
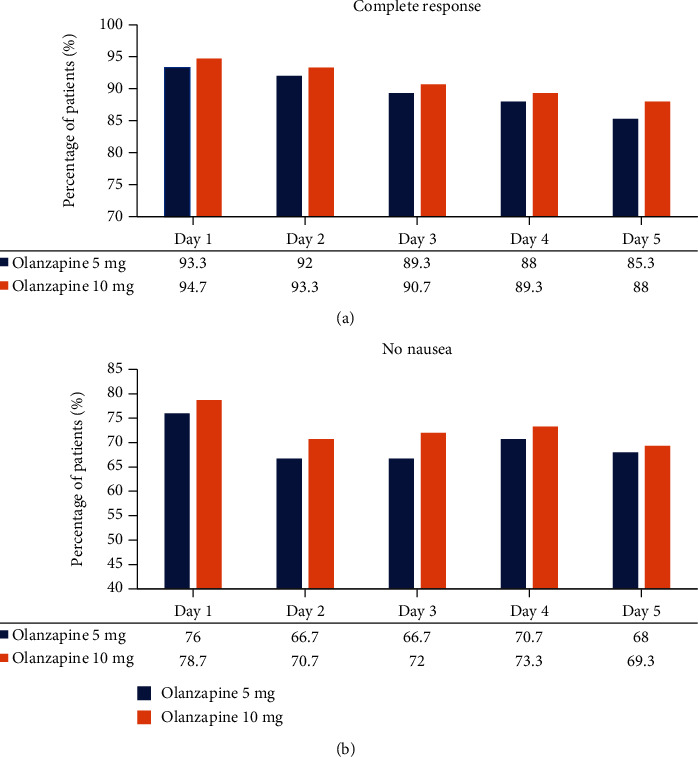
The incidence of complete response (a) and no nausea (b) in olanzapine 5 mg and 10 mg groups from day 1 (the initiation of cisplatin) to day 5.

In the olanzapine 5 mg group, the proportions of CR were 93.3%, 92%, 89.3%, 88%, and 85.3% in day 1 daily to day 5, respectively, and the incidence of no nausea was 76%, 66.7%, 66.7%, 70.7%, and 68% in day 1 daily to day 5, respectively (Figure 1). In the inolanzapine 10 mg group, the proportions of CR were 94.7%, 93.3%, 90.7%, 89.3%, and 88% in day 1 to day 5, respectively, and the incidence of no nausea were 78.7%, 70.7%, 72%, 73.3%, and 69.3% from day 1 to day 5, respectively (Figure 1).

In addition, the proportions of CR in the olanzapine 5 mg group were comparable to those in the olanzapine 10 mg group in acute (93.3% [*n* = 70] vs. 94.7% [*n* = 71], *P* = 0.731), delayed (76% [*n* = 57] vs. 78.7% [*n* = 59], *P* = 0.697), and overall phase (73.3% [*n* = 55] vs. 77.3% [*n* = 58], *P* = 0.570) (Table 2). Regarding to the secondary end point (no nausea), the proportions of no nausea were also comparable between the two groups in acute (76% [*n* = 57] vs. 78.7% [*n* = 59], *P* = 0.697), delayed (54.7% [*n* = 41] vs. 60% [*n* = 45], *P* = 0.509), and overall period (50.7% [*n* = 38] vs. 57.3% [*n* = 43], *P* = 0.111) (Table 2).

### 3.3. Adverse Events

We evaluated the common adverse effects (hiccups, somnolence, and constipation) in the treatment of olanzapine. No patients discontinued olanzapine due to treatment-related adverse events. During 0-120 hours after the initiation of olanzapine, somnolence (50.0%, *n* = 75) was the most common adverse effect in the whole cohort, followed by constipation (22.0%, *n* = 33) and hiccups (10.0%, *n* = 15). The majority of the side effects were grade 1 and grade 2, and only one patient suffered grade 3 somnolence. The incidence of somnolence in the olanzapine 10 mg group (58.6%) was significantly higher than that in the olanzapine 5 mg group (41.3%) (*P* = 0.034), while constipation (20.0% vs. 24.0%, *P* = 0.554) and hiccups (9.3% vs. 10.6%, *P* = 0.785) rates were comparable in two groups. The details of adverse effect distribution in two experimental groups were presented in Table 3.

## 4. Discussion

To the best of our knowledge, this study was the first to explore the optimum dose of olanzapine (5 mg or 10 mg) combined with triple combination antiemetic therapy against CCRT-induced nausea and vomiting in NPC patients. The result showed that patients receiving olanzapine 10 mg had a comparable antiemetic effect, but a higher incidence of adverse effects (especially somnolence) compared with those treated with olanzapine 5 mg whether in acute, delayed, or overall phases.

Numerous previous studies had elaborated that the combination of olanzapine 5 mg or 10 mg antiemetic therapy provided a significant improvement against highly emetogenic chemotherapy [14, 16, 20, 21]. However, rare studies compared the efficacy of olanzapine 5 mg and 10 mg in preventing cisplatin-based CINV. A multi-institutional phase II study from Japan including 153 patients (76 in 10 mg group, 77 in the 5 mg group) showed that the CR rate in the 5 mg group was significantly higher than that in the 10 mg group during the delayed phase (85.7% vs. 77.6%, *P* < 0.05) [22]. Their results were different from our study in that the proportion of achieving CR in the 10 mg group was higher than that in the 5 mg group; yet, there was no difference (76% vs. 78.7%, *P* = 0.697). In addition, the CR rates in acute and overall phases in their study were also higher than those in the present study [22]. The possible explanation was that all the included participants in this study were NPC patients who were receiving irradiation of nasopharynx and cervical lymph nodes and cisplatin-based chemotherapy, which made them easier to suffer vomiting than other cancer patients [22]. Additionally, the dose of cisplatin in their study (mean dose 72.9 mg/m^2^) was lower than that in our study (100 mg/m^2^), which could still result in a higher CR rate.

No nausea rate was another effective assessment for the efficacy of antiemetic drugs. In the present study, the percentage of no nausea in the olanzapine 5 mg group was 76%, 54.7%, and 50.7% in acute, delayed, and overall periods, respectively, which were higher than the result from a phase 3 randomized trial conducted by Navari et al. that 73.8% in the acute phase, 42.4% in the delayed phase, and 37.3% in overall phase in the olanzapine 5 mg group [17]. It is worth noting that 89.6% of the patients were of the white race, and only 2.6% were Asians in their study, while all of the patients in our study were Asians. Previous studies have shown that race might have an association with different levels of CINV [23, 24]; hence, the distribution of race might be the reason for the difference. Another study from Navari et al. explored the effect of olanzapine (10 mg), palonosetron, dexamethasone compared to fosaprepitant, palonosetron, and dexamethasone on CCRT-induced nausea and vomiting in locally advanced head and neck or esophageal cancer. They found that CR rate was similar between the treatment arms, while nausea in the delayed and overall periods was significantly improved in those treated with olanzapine compared to those without olanzapine [25]. It is worth noting that triplet-combination regimen with olanzapine 10 mg in the above study had higher no nausea rates in acute (86% vs. 78.7%), delayed (71% vs. 60%), and overall periods (71% vs. 57.3%) compared with four-drug combination regimen with olanzapine 10 mg in our study. The reason might be that the dose of cisplatin they used was lower than ours in CCRT (> 70 mg/m^2^ vs. 100 mg/m^2^), making the no nausea rates higher than our study [25]. In addition, the proportions of no nausea were comparable between olanzapine 5 mg and 10 mg groups no matter in acute (*P* = 0.69), delayed (*P* = 0.51), and overall period (*P* = 0.11) in our study. Therefore, a four-drug combination regimen with olanzapine 5 mg might be enough in the control of nausea in NPC patients receiving CCRT.

Somnolence is the most common adverse effect associated with olanzapine. Previous studies have shown that sleepiness remained a matter of serious concern in 53.3%-73% of the patients receiving olanzapine 10 mg, which was higher than those treated with olanzapine 5 mg (22.7%-45.5%) [26–28]. In the present study, the incidence of somnolence in olanzapine 5 mg and 10 mg groups was 58.6% and 41.3%, respectively. Among them, grade 1 and grade 2 somnolence in the olanzapine 10 mg group was all higher than that in the olanzapine 5 mg group, and 1 patient receiving olanzapine 10 mg suffered grade 3 somnolence. Our result is consistent with aforementioned studies [22, 26, 27]. To our knowledge, drowsiness greatly impacts the patients' daily life, including communication, social function, and sleep quality [28, 29]; therefore, based on equivalent antiemetic effect and lower adverse effect, olanzapine 5 mg plus triplet-combination may become the standard antiemetic regimen in NPC patients receiving cisplatin-based CCRT.

Several limitations should be noted in this study. Firstly, 62% of the patients have received 2-3 cycles of IC before the initiation of randomization, and higher cumulative chemotherapy dose seems to be more prone to nausea and vomiting, which might affect the accuracy of the results [30]. Secondly, this study was from a single healthcare system, and all patients were Chinese. Previous studies have shown that race might be associated with different levels of CINV [23, 24]. Hence, large-scale and multicenter studies should be carried out if the result would be fully applicable to all the patients.

In conclusion, this study suggests that patients receiving olanzapine plus standard antiemetic therapy have excellent antiemetic effects in NPC patients receiving cisplatin-based CCRT, and olanzapine 5 mg has a similar antiemetic effect and lower adverse effects (somnolence) compared with those with olanzapine 10 mg. Therefore, olanzapine 5 mg may serve as the standard antiemetic regimen in NPC patients receiving cisplatin-based CCRT.

## Figures and Tables

**Table 1:** Characteristics of the study patients (*N* = 88).

Variables	*N*	%
Gender		
Male	68	77.3
Female	20	22.7
Age (year)		
Mean	46	
Range	23-68	
Smoking history		
Yes	50	56.8
No	38	43.2
Alcohol consumption		
Yes	22	25
No	66	75
Pathological type		
Undifferentiated non-keratinizing carcinoma	76	86.4
Differentiated non-keratinizing carcinoma	9	10.2
Mixed type of above two	3	3.4
Clinical stage		
II	14	15.9
III	36	40.9
IVa	33	37.5
IVb	5	5.7
T stage		
T1	12	13.6
T2	20	22.7
T3	40	45.5
T4	16	18.2
N stage		
N0	4	4.6
N1	33	37.5
N2	28	31.8
N3	23	26.1
M stage		
M0	83	94.3
M1	5	5.7
Induction chemotherapy		
Yes	62	70.5
No	26	29.5

**Table 2:** The complete response and no nausea rate and in acute, delayed, and overall phase between two groups.

Variables	Olanzapine 5 mg	Olanzapine 10 mg	*P*
Acute phase (0-24 h)			
Complete response	70 (93.3%)	71 (94.7%)	0.731
No nausea	57 (76%)	59 (78.7%)	0.697
Delayed phase (25–120 h)			
Complete response	57 (76%)	59 (78.7%)	0.697
No nausea	41 (54.7%)	45 (60%)	0.509
Overall phase (0–120 h)			
Complete response	55 (73.3%)	58 (77.3%)	0.570
No nausea	38 (50.7%)	43 (57.3)	0.111

**Table 3:** Treatment-related adverse events in the study population.

Variables	Olanzapine 5 mg			Olanzapine 10 mg			*P*
	Grade 1	Grade 2	Grade 3	Grade 1	Grade 2	Grade 3	
Somnolence	26 (34.7%)	5 (6.7%)	0	34 (45.3%)	9 (12%)	1 (1.3%)	0.034
Constipation	13 (17.3%)	2 (2.7%)	0	14 (18.7%)	4 (5.3%)	0	0.554
Hiccups	6 (8%)	1 (1.3%)	0	7 (9.3%)	1 (1.3%)	0	0.785

## Data Availability

The raw data supporting the conclusions of this article is available from the corresponding authors on reasonable request.
